# Supplementary figures and images for: Study protocol of guided mobile-based perinatal mindfulness intervention (GMBPMI) - a randomized controlled trial
Source: PLoS One. 2022 Jul 8;17(7):e0270683. doi: 10.1371/journal.pone.0270683 (PMC9269359; doi:10.1371/journal.pone.0270683)

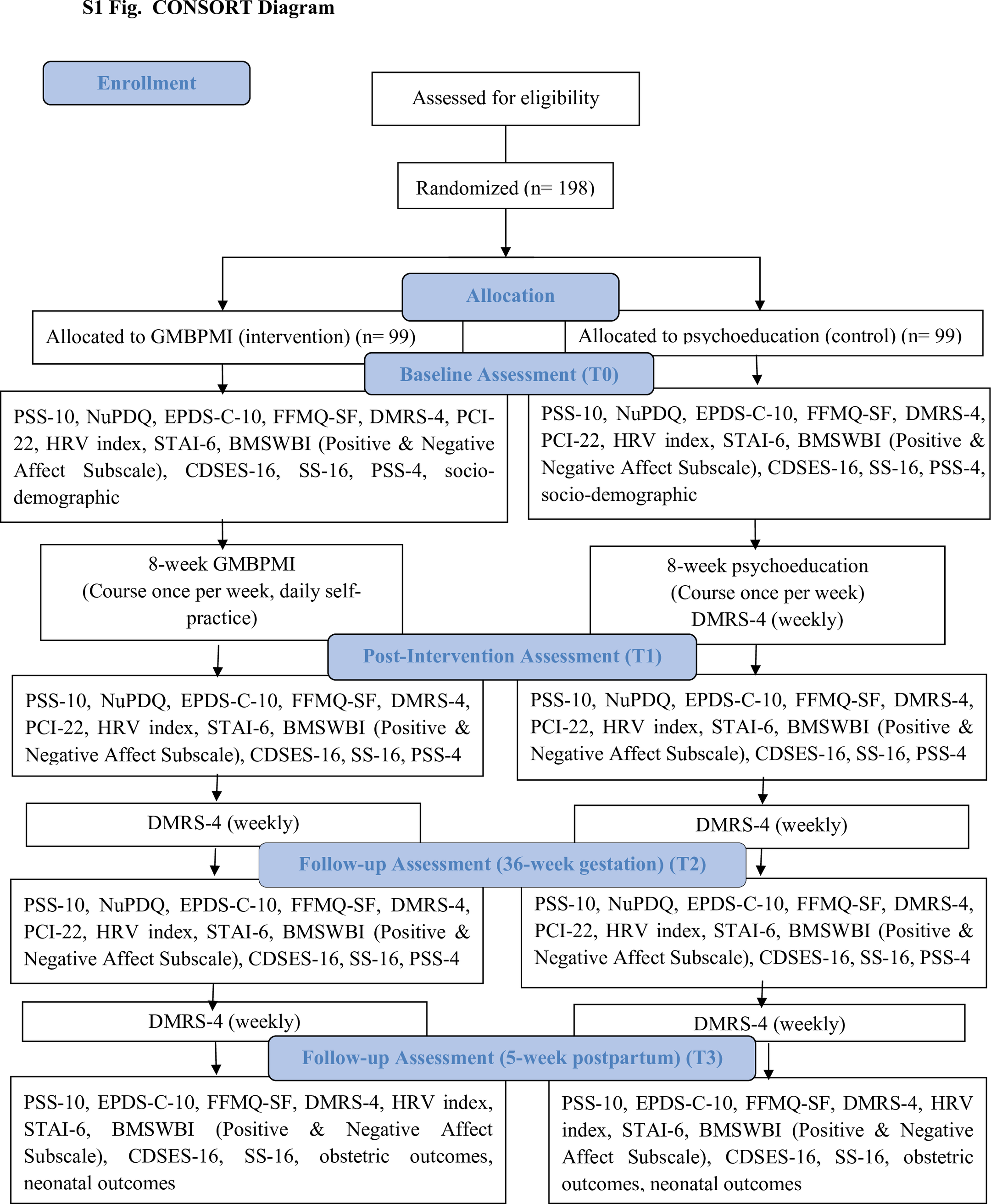

Supplement: S1 Fig — (TIFF) [file pone.0270683.s001.tiff]
