# Supplementary material for: Study protocol of guided mobile-based perinatal mindfulness intervention (GMBPMI) - a randomized controlled trial
Source: PLoS One. 2022 Jul 8;17(7):e0270683. doi: 10.1371/journal.pone.0270683 (PMC9269359; doi:10.1371/journal.pone.0270683)
Supplement: S1 File — (DOCX) [file pone.0270683.s002.docx]

**A Guided Mobile-Based Perinatal Mindfulness Intervention - A Randomized Controlled Trial**

**BACKGROUND OF RESEARCH**

***Contemporary Expectant Mothers in Hong Kong Facing Tremendous Psychological Stress***

Being pregnant, giving birth and turning into a parent are three closely intertwined major life transitions that a woman would experience within a short period of time. For many women, these transitions are treasured. In parallel this period can be clouded by significant psychological distress, due to unparalleled emotional, physical, and interpersonal changes naturally occurred (Howard, Piot, & Stein, 2014). Pregnant women face a set of stresses, including heightened health and medical concerns, physical discomfort, difficulty in navigating new identify, fear and anxiety associated with childbirth, and self-critical thought of not being a “perfect mother” (Byatt et al., 2013; Dunkel Schetter, 2011; Kingston et al., 2015).

In Hong Kong, contemporary women face added pressures resulting from living in a competitive, work-intense society. Women’s labor force participation rate is high (84.1% in the 20-39 age group) and Hong Kong ranks among the fifth with the oldest first-time mother in the world (median age 31.6 years old; Hong Kong Census and Statistics Department, 2019). Pregnancy at an older age is a known risk factor for antenatal depression (Zeng, Cui, & Li, 2015). The difficulty of balancing work, home and self-care may be amplified during pregnancy (Goodman & Crouter, 2009). Furthermore, pregnant women in Hong Kong face the stress of negotiating through cultural traditions. Cultural rituals of maternal care are still commonly practiced, such as conforming to an array of dietary and behavioral proscriptions (Lee et al., 2009). This could be a source of conflict between a pregnant women and her significant others (Leung, Lee, Chiang, Lam, et al., 2013). Relationship tension is a risk factor for perinatal depression (Lau & Keung, 2007; Siu, Leung, Ip, Hung, & O'Hara, 2012).

On the other hand, prenatal period represents an excellent opportunity to engage and strengthen women’s stress management and coping efficacy (Guardino & Dunkel Schetter, 2014). Pregnant women are often highly motivated to seek help for their babies’ well-being, making preventive interventions much more acceptable (Woolhouse, Brown, Krasteve, Perlen & Gunn, 2009).

***Adverse Consequences of Maternal Psychological Stress (MPS)***

MPS during perinatal period is associated with broad and profound adverse consequences for both the mother and child, making it an important public health issue (Halbreich, 2005). Accumulating evidences suggest association between MPS and unfavorable obstetrics and neonatal outcomes (Field, Diego, & Hernandez-Reif, 2006). These include obstetric complications, such as increased analgesic use and unplanned caesarean delivery (Alder et al., 2007; Martini et al., 2010). Fetus’s exposure to MPS increases his/her risk of unfavorable neonatal results, including preterm delivery (Lau, 2013), low birth weight (Dunkel Schetter, 2011), low Apgar scores, smaller head circumference and major congenital anomalies (Marcus, 2009; Räisänen et al., 2014).

Research started to reveal that the disrupting maternal cardiovascular system in pregnant women with elevated MPS, indexed by low heart rate variability (HRV), might be a mechanism for the worse birth outcomes. HRV is an index of parasympathetic control and autonomic flexibility, indicating cardiovascular ‘lability’ (Porges, 2007). Higher HRV suggests parasympathetic activation and flexibility of autonomic regulation. Lowered HRV implies compromised cardiovascular responsiveness (Ecklund-Flores et al., 2017). Shea (2008) revealed association of lower HRV among pregnant women with elevated prenatal depressive symptoms and worse birth outcomes. However, this is not conclusive and further in-depth studies in this area are awaited.

***Mindfulness-Based Interventions (MBI) Holding Promises to Protect Pregnant Women from Stress***

MBI may be uniquely suitable and beneficial for women during perinatal period. Mindfulness practice, which facilitates greater awareness and acceptance of one’s present moment sensations, emotions and thoughts, may be conducive for expectant mothers to effectively face, process, relate to and cope with physical discomfort and negative emotions or thoughts arose during perinatal period (Bonacquisti, Cohen, & Schiller, 2017; Hughes et al., 2009). Mindfulness practice cultivates abilities important to pregnant women and new mothers, such as savoring, self-acceptance and psychological flexibility.

In the recent decade, empirical researches suggesting the efficacy of prenatal MBI are emerging. For example, Zhang (2018) conducted a randomized controlled trial with 66 Chinese pregnant women and revealed the efficacy of MBI in reducing prenatal stress. In a meta-analysis of 17 studies of prenatal MBI, MBIs demonstrated significant pre/post improvements of medium effect size for reducing stress and depression (Taylor, Cavanagh & Strauss, 2016). However, at postnatal, there were no significant improvements in stress and depression, and in mindfulness skills for MBI participants versus controls (Taylor, Cavanagh & Strauss, 2016). For practical reasons, existing MBIs focused on the second trimester of pregnancy. Findings suggest a need to extend the intervention to the third trimester and postnatal.

Previous studies have shown that MBI led to increased HRV, and decreased blood pressure and cortisol levels (Carlson, Speca, Faris, & Patell 2007; Shearer, Hunt, Chowdbury, & Nicole, 2016), suggesting that MBI may have beneficial physiological effects relevant to pregnant women (Braeken, Jones, Otte, Nyklíček, & Van den Bergh, 2017). Importantly, MBI may also increase a mother’s odd of having a healthy child (Isgut et al., 2017). Higher maternal mindfulness of the women at 22 weeks gestation significantly predicted a normal neonatal birth weight, in contrast to low birth weight (Nyklíček, Truijens, Spek, & Pop, 2018). Van den Heuvel and colleagues (2015a, 2015b) showed that maternal mindfulness was negatively correlated with maternal anxiety during pregnancy, and positively associated with less infant self-regulation problems, less difficult temperament and more effortful control of infants at ten months old.

***The Calling for Technological Innovation in Providing Perinatal Mental Health Care***

Research has shown that fewer than half of pregnant women in distress will seek help (Goodman & Tyer-Viola, 2010). A number of barriers prevent them from engaging with professional services, including physical changes, burden of childrearing and household chores/work (Loughnan, Newby, Haskelberg, Mahoney, et al., 2018). Women are also stressed by perceived social pressure to be a strong mother and to sacrifice personal welfare for the sake of her baby (Woolhouse et al., 2009). An approach to greatly widen the access of service to pregnant women is using mobile-based interventions (Spijkerman, Pots & Bohlmeijer, 2016). Allowing practice at home and flexibility in time schedule are perfect responses to the predicaments of pregnant women and new mothers (Andrew, Cuijpers, Craske, McEvoy, & Titov, 2010). Moreover, mobile-based interventions are scalable at a reduced cost, making it a potential solution to a major public health concern.

***What We Have Done – The Eastern-Based Meditation Intervention (EBMI) for Chinese Pregnant Women and the Guided Mobile-Based Perinatal Mindfulness Intervention (GMBPMI)***

In Hong Kong, we developed and evaluated a cultural-sensitive MBI - Eastern-Based Meditation Intervention (EBMI) for pregnant women (Chan, 2015). EBMI was theorized to help reduce perinatal psychological stress through continued meditation practices and cultivation of mindfulness. The efficacy of EBMI has been evaluated with 123 pregnant women by a randomized controlled trial (Chan, 2014, 2016). Comparing with control group, participants in EBMI group who practiced meditation regularly showed significantly higher positive appraisal in face of prenatal distress, and lower levels of “stress hormone”– salivary cortisol in the evening, suggesting a restored balance of the hypothalamic-pituitary-adrenal axis neuroendocrine function. Infant outcomes were also promising, including higher level of cord blood cortisol. High cord blood cortisol levels are believed to protect against the development of neonatal health abnormality, for example, respiratory morbidities (Tsuda et al., 2016). Babies from the intervention group also displayed less difficult temperament at 5-month old. They were more willing to approach novel things and were less moody.

However, the significant effects were only observed among those 56% participants who practiced mindfulness regularly (Chan, 2015). Lacking continued motivation was the common reason reported for giving up the practice. To overcome the limitations of the face-to-face EBMI, we developed a guided mobile-based perinatal mindfulness intervention (GMBPMI). To increase its accessibility, the 6 weekly sessions of EBMI were put on the web. With the guidance of a trained Research Assistant (RA), pregnant women can complete these sessions flexibly at home during the second trimester. Support for the pregnant women continues through the third trimester up to 5 weeks postpartum. Through social media platform (WhatsApp or WeChat), the RA sends out prompt and link for mindfulness practice daily, and initiates chat every week. The chat is around experiences/ difficulties in mindfulness practice. The RA is backed up by the PI and co-I’s who are experienced mental health practitioners and mindfulness teachers. One of them is an experienced obstetrics and gynaecology specialist.

In the past year, we piloted GMBPMI with three pregnant women and refined the protocol. The feasibility and acceptability of the intervention seemed good, as evidenced by participants’ positive feedback and high compliance to daily mindfulness practice (around 80% to 90%). They welcome and valued the continued support of the RA via social media platform.

**REASEARCH PLAN AND METHODOLOGY**

***Research Gap and Significance of the Proposed Study***

While the efficacy of prenatal MBI has been revealed in the past decade, the availability and usage of the programme remain low for various practical reasons depicted earlier. Moreover, providing only a limited number of lessons during the prenatal period seems insufficient. It is highly desirable to provide extended support so as to ensure continued mindfulness practice in both the pre- and post-natal period. To address these issues, a guided mobile-based intervention appears to be a good choice. In Hong Kong, around 50,000 newborn babies are delivered each year. To meet the service needs of this scale, it is imperative for us to develop a more cost-effective, accessible and acceptable intervention. In light of the promising findings of our earlier studies, GMBPMI may be a practical solution. The proposed study aims to rigorously evaluate the efficacy of GMBPMI.

***Conceptual Framework of the Research***

The study adopts the transactional theory of stress and coping as the underpinning theoretical framework. Transactional theory proposes how people react to stress, and the intensity of such reaction is heavily influenced by the mediating role of appraisal, the cognitive process through which meaning is ascribed to the events (Boyd, Lewin, and Sager, 2009; Dewe and Cooper, 2007). By reordering life priorities based upon one’s values and goals and ascribing positive meaning to stressful event, meaning-focused coping helps restore the resources that influence cognitive appraisals, sustain coping efforts over time, and provide relief from distress (Carver & Connor-Smith, 2010; Folkman, 2008).

Through continued mindfulness practices, perinatal women can increase their maternal mindfulness. Increased maternal mindfulness can expand pregnant women’s perceptual awareness and facilitate positive appraisal during stressful perinatal period, thereby help to reduce perinatal psychological stress. This is consistent with the results of our previous studies, where positive appraisal was found significantly increased in mindfulness group (Chan, 2015). Moreover, by reducing perinatal psychological stress, the increase of maternal mindfulness is theorized to lead to higher HRV, suggesting increased parasympathetic control. The decreased perinatal psychological stress can bring about better birth outcomes (obstetric and neonatal), through increased parasympathetic activity. Figure 1 depicts this conceptual model.


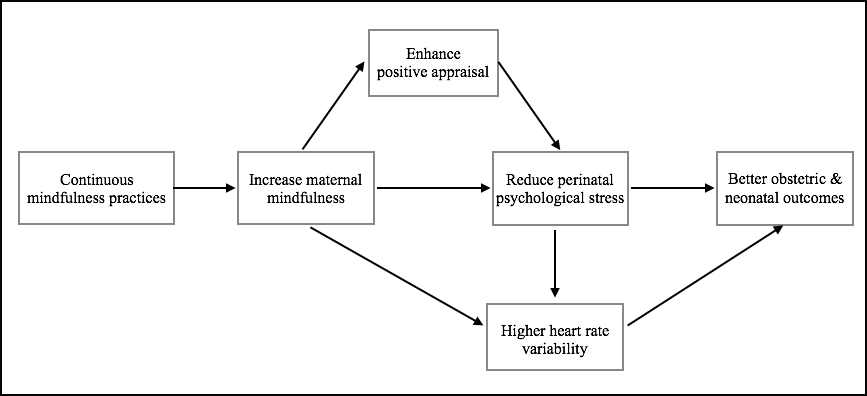


Figure 1. Conceptual Framework

***Research Design, Objectives and Hypotheses***

The research adopts a parallel-armed, randomized controlled trial (RCT) design. Participants will be pregnant women in the second trimester. Measures will be taken at 4 time-points: pre-intervention (T0), completion of the 6 weekly online lessons of EBMI in intervention group or 6 weekly online lessons of psychoeducation on perinatal care in control group (T1), 36 weeks gestation (T2), and 5 weeks postpartum (T3)

The objectives are:

1. To evaluate the efficacy of the intervention in terms of maternal mindfulness and positive appraisal;
2. To evaluate the efficacy of the intervention in terms of general and pregnant related stress and depression;
3. To evaluate the efficacy of the intervention in terms of HRV;
4. To examine the trajectory of general and pregnant related stresses in relation to obstetric and neonatal outcomes at 5 weeks postpartum period.

Hypotheses are:

1. The intervention group would show greater increase in maternal mindfulness and positive appraisal than control group at T1, T2 and T3;
2. The intervention group would show greater reduction in stress and depression than control group at T1, T2 and T3, and these outcomes would be mediated by the enhancement in positive appraisal;
3. The intervention group would show greater increase in HRV than the control group at T1, T2 and T3, and these outcomes would be mediated by the reduction in stress and depression;
4. The level of stress and depression would be negatively associated with obstetric and neonatal outcomes at T3, and these correlations would be mediated by HRV.

***Participants and Sample Size Calculation***

Previous EBMI study reported an effect size of 0.44 in reducing prenatal distress and attrition rate at around 20%. In the proposed study, we assume an overall moderate effect size of 0.4 and a conservative attrition rate of 25%. Setting a power at 90%, significance level of *p*< .008 (corrected for 6 primary outcomes), a sample size of 99 per arm is needed for 2-arm repeated measures design (GPower, version 3.1). Thus, the targeted total sample size is 198.

***Measures***

Measures of the related variables are summarized in Table 1. In line with the study’s objectives, primary outcome variables are general stress, pregnant related stress, depression, mindfulness, positive appraisal and HRV. Other variables measuring psychological and physical well-being are incorporated as the secondary outcome variables, including anxiety, affect, stagnation and spirituality. Also included are a group of birth outcomes, including obstetric and neonatal outcomes. Additionally, for the purpose of controlling potential confounding effects, risk factors documented in the literature will be measured, including an array of socio-demographic factors and social support.

Table 1. Outcome variables

| **Variables** | **Measures** | **Timepoints** |
| --- | --- | --- |
| **Primary outcome variables** | | |
| Maternal psychological stress | | |
| General stress | Perceived Stress Scale -10 items – The scale measures subjective perception of stress that includes two subscales, perceived helplessness and perceived self-efficacy (Cohen & Williamson, 1988; Ng, 2013). | T0, T1, T2, T3, |
| Pregnancy specific stress | Prenatal Distress Questionnaire – 12 items – The scale consists 3 factors, which are concerns about birth and baby, weight and body image, and emotions and relationships (Alderdice & Lynn, 2011). | T0, T1, T2 |
| Depression | Edinburgh Postnatal Depression Scale – Chinese – 10 items – The scale has been validated in Chinese population with satisfactory psychometric properties (Lee, Yip, Chiu, Leung, et al., 1998). | T0, T1, T2, T3 |
| Mindfulness | | |
| State mindfulness | Short-form Five Facet Mindfulness Questionnaire – 20 items – The scale consists 5 subscales – observing, describing, acting awareness, non-judging to inner experience and non-reacting to inner experience (Hou, Wong, Lo, Mak, & Ma, 2013). | T0, T1, T2, T3 |
| Daily mindfulness | Daily Mindful Responding Scale – 4 items – It is a measure designed to assess mindful responding in the daily lives of people undergoing mindfulness training (Lacaille, et al., 2015). | Every week until T3 |
| Positive appraisal | | |
| Coping | Prenatal Coping Inventory – 22 items – It consists 4 subscales, namely preparation, avoidance, positive appraisal, and prayer (Lobel et al., 2002). | T0, T1, T2 |
| Heart rate variability | | |
|  | Electrocardiogram Mobile Monitor by Karda – It is a FDA cleared wearable device monitors electrocardiogram. | T0, T1, T2, T3 |
| **Secondary outcome variables** | | |
| Psychological well-being | | |
| Anxiety | Short-form State subscale of the State-Trait Anxiety Inventory – 6 items - The scale has been validated in pregnant women with satisfactory psychometric properties (Marteau &Bekker, 1992). | T0, T1, T2, T3, |
| Affect | Positive & Negative Affect Subscales of Body-Mind-Spirit Well-being Inventory – 8 and 16 items - The two subscales measure common positive and negative affect in Chinese adults (Ng et al., 2005). | T0, T1, T2, T3, |
| Spirituality | Chinese Daily Spiritual Experience Scale – 16 items – The scale measures a person’s perception of involvement of the transcendence in daily life (Ng et al., 2009; Underwood & Teresi, 2002). | T0, T1, T2, T3, |
| Physical well-being | | |
| Stagnation | Stagnation Scale – 16 items - The scale measures a cluster of mind/body obstruction-like symptoms depicted in Chinese medicine. It has 3 factors, namely body/mind obstruction, affect/posture inhibition and overattachment (Ng et al., 2006; Ng et al., 2012). Stagnation was identified as a mediator in explaining the mechanism of change in mindfulness (Lo et al., 2013). | T0, T1, T2, T3, |
| **Clinical outcomes** | | |
| Obstetric outcomes | Gestational age at birth, pregnancy complications, mode of birth. | T3 |
| Neonatal outcomes | Birth weight, head circumference, months of maturity, and Apgar score. | T3 |
| **Individual factors** | | |
| Socio-demographic | Age, gender, marital status, family income, employment, education, gestational age, parity, obstetric history, medical history, and pre-pregnancy BMI. | T0 |
| Social support | Prenatal Social Support – 4 items – The scale measures the level of social support women receive during pregnancy (Collins et al., 1993). | T0, T1, T2 |

***Sampling, Recruitment, Randomization, and Procedure***

Adult (age 18 or above) pregnant Chinese women in their 2^nd^ trimester (between 12^th^ to 20^th^ week gestation) will be invited to the study. This is when pregnancy becomes stable. Participants will be recruited from the community through advertisements posted on relevant websites, maternal discussion forums, Facebook, and Twitter. The exclusion criteria are: 1) not able to understand Chinese (the intervention will be delivered in Chinese); 2) high-risk pregnancy status (e.g., preterm labor, placental abnormality, multiple gestations, required bed rest, or morbid obesity); and 3) current psychiatric disorders that necessitated priority attention (e.g., schizoaffective disorder, bipolar disorder, or current psychosis; organic mental disorder or pervasive developmental delay; current substance abuse or dependence; imminent suicide or homicide risk). Appropriate referral to other resources will be made carefully if the pregnant woman needs help.

When a potential participant contacted and expressed interests, an online/phone intake interview will be conducted by the project RA. The intake aims to serve 3 purposes: 1) assessing eligibility; if eligible 2) explaining the study in detail and addressing concerns; and 3) seeking informed consent. The participant will receive a consent form by email and will be asked to return it back to the researchers. Subsequently a mobile device for monitoring HRV – KardaMobile ECG will be mailed to each participant.

Because the participants’ recruitment will proceed on a continual basis, once it starts, random allocation process will be performed weekly. Every week, each participant recruited within that week will be randomly allocated to either the experimental or control groups on a 1-to-1 ratio according to a computer-generated random digit. Since the project RA is responsible for delivering the interventions, another RA will handle data collection, primarily by web-based software (Google Form). Participants’ personal identifiers will not be included in the dataset.

***Experimental and Control Group***

Participants in the experimental group will receive GMBPMI. A new participant (a pregnant woman in the second trimester) is expected to complete the 6 EBMI lessons in 6 weeks, and do the mindfulness practice for about 30-60 minutes daily. The project RA will send prompt and guidance for daily mindfulness practice to each participant through social media platform. Participants will also be asked to keep log of daily mindfulness practice from T0 to T3 using Google Form. The project RA will be available online to support, and will initiate chat every week throughout the whole intervention period. The chats will focus on participants’ experiences or difficulties of mindfulness practice. The RA is backed up by the PI and co-I’s who are experienced mental health practitioner and mindfulness teacher. One of them is an obstetrics and gynaecology specialist.

To control for attention and placebo effects, every new participant in the control group will receive weekly web-based psychoeducation program for perinatal care. The project RA will also be available online to support, and will initiate chat every week throughout the whole intervention period. The chats will focus on participants’ experiences of the psychoeducation program.

***Data Analysis***

Intention-to-treat analysis will be performed, and missing data due to drop–out will be handled by the ‘last observations carried forward’ principle. 1) With a 2-arm RCT design, the efficacy of the intervention will be examined by repeated measures MANOVA on both primary and secondary outcome variables, while adjusting for individual factors. Partial eta-squared values will be calculated to measure the effect size; values of .02, .13, and .26 suggest small, medium, and large effect sizes respectively (Pierce, Block, & Aguinis, 2004). 2) To investigate within group effect, serial trend analysis from T0 to T3 will be conducted on the primary and secondary outcome variables. 3) A series of linear mixed-effects models will be fitted to assess the mediation effects. 4) For HRV data, the software Kubios will be used for data extraction and reduction. Three HRV indexes will be computed for analysis, namely RMSSD (Mean squared difference between consecutive normal-to-normal intervals), SDNN (standard deviation of beat-to-beat intervals), and HF (absolute power of HF band).
