# Supplementary material for: Study protocol of guided mobile-based perinatal mindfulness intervention (GMBPMI) - a randomized controlled trial
Source: PLoS One. 2022 Jul 8;17(7):e0270683. doi: 10.1371/journal.pone.0270683 (PMC9269359; doi:10.1371/journal.pone.0270683)
Supplement: S3 File — (PDF) [file pone.0270683.s004.pdf]

S3 File. All items from the World Health Organization Trial Registration Data Set

Data Category	Information
Primary registry and trial identifying number	ClinicalTrials.gov: NCT04876014
Date of registration in primary registry	March 30, 2021
Secondary identifying numbers	17603520
Source of monetary or material support	The University of Hong Kong
Primary sponsor	The University of Hong Kong
Secondary sponsor	The University of Hong Kong
Contact for scientific queries	Prof. Siu-man Ng [(852) 3917 4370] [ngsiuman@hku.hk]
Public title	A Guided Mobile-Based Perinatal Mindfulness Intervention
Scientific title	A Guided Mobile-Based Perinatal Mindfulness Intervention – A Randomized Controlled Trial
Countries of recruitment	Hong Kong SAR, China
Health conditions or problems studied	Maternal psychological distress<br>Mental health wellness
Intervention	Guided Mobile-Based Perinatal Mindfulness Intervention<br>GMBPMI includes 6 EBMI lessons in 6 weeks, and it will require the participants to do mindfulness practice for about 30-60 minutes daily. The project RA will send prompt and guidance for daily mindfulness practice to each participant through social media platform. The RA will be available online to support, and will initiate chat every week throughout the whole intervention period. The chats will focus on participants' experiences or difficulties of mindfulness practice.
Key inclusion and exclusion criteria	Inclusion Criteria:<br>• Adult age 18 or above<br>• Pregnant Chinese women in their 2nd trimester<br>Exclusion Criteria:<br>• Not able to understand Chinese (the intervention will be delivered in Chinese)<br>• High-risk pregnancy status (e.g., preterm labor, placental abnormality, multiple gestations, required bed rest, or morbid obesity)<br>• Current psychiatric disorders that necessitated priority attention (e.g., schizoaffective disorder, bipolar disorder, or current

	psychosis; organic mental disorder or pervasive developmental delay; current substance abuse or dependence; imminent suicide or homicide risk)
Study type	Interventional
Date of first enrolment	May 7, 2021
Target sample size	198
Recruitment status	Active, recruiting
Primary outcomes	• Maternal psychological stress-General Stress ('change' is being assessed) [Time Frame: Change from baseline General Stress at post intervention (8 weeks), 36-week gestation and 5-week postpartum.]<br><br>General stress will be measured by Perceived Stress Scale -10. The minimum and maximum values for each item is 1 to 4 with lower score represents lower stress.<br><br>• Maternal psychological stress-Pregnancy specific stress ('change' is being assessed) [Time Frame: Change from baseline Pregnancy specific stress at post intervention (8 weeks) and 36-week gestation.]<br><br>Pregnancy specific stress will be measured by Prenatal Distress Questionnaire-12. The minimum and maximum values for each item are 0 to 4, with lower score represents lower stress.<br><br>• Maternal psychological stress-Depression ('change' is being assessed) [Time Frame: Change from baseline Depression at post intervention (8 weeks), 36-week gestation and 5-week postpartum.]<br><br>Depression is measured by Edinburgh Postnatal Depression Scale-Chinese-10. The minimum and maximum values are 1 to 4, with higher score represents higher depressive symptoms.<br><br>• Mindfulness-State mindfulness ('change' is being assessed) [Time Frame: Change from baseline State MIndfulness at post intervention (8 weeks), 36-week gestation and 5-week postpartum.]<br><br>State mindfulness is measured bu Short-form Five Facet Mindfulness Questionnaire-Chinese-20 items. The minimum and maximum values are 0 to 4, with higher score represents higher state of mindfulness.

	• Mindfulness-Daily mindfulness ('change' is being assessed) [Time Frame: Weekly changes from baseline Daily Mindfulness at 5-week postpartum.]<br><br>Daily mindfulness is measured by Daily Mindful Responding Scale - 4 items. The minimum and maximum values are 0 to 10, with higher score represents higher state of mindfulness.<br><br>• Positive appraisal-Coping ('change' is being assessed) [Time Frame: Change from baseline Coping at post intervention (8 weeks) and 36-week gestation.]<br><br>Coping is measured by Prenatal Coping Inventory - 22 items. The minimum and maximum values are 0 to 4, with higher score represents higher coping frequency.<br><br>• Heart rate variability ('change' is being assessed) [Time Frame: Change from baseline HRV at post intervention (8 weeks), 36-week gestation and 5-week postpartum.]<br><br>HRV score, with higher score represents higher physical and mental condition for the day.
Key secondary outcomes	• Psychological Well-being- Anxiety ('change' is being assessed) [Time Frame: Change from baseline Anxiety at post intervention (8 weeks), 36-week gestation and 5-week postpartum.]<br><br>Anxiety is measured by Short-form State subscale of the State-Trait Anxiety Inventory - 6 items. The minimum and maximum values range from 0 to 4, with higher score represents higher anxiety level.<br><br>• Psychological Well-being- Affect ('change' is being assessed) [Time Frame: Change from baseline Affect at post intervention (8 weeks), 36-week gestation and 5-week postpartum.]<br><br>Affect is measured by Positive & Negative Affect Subscales of Body-Mind-Spirit Well-being Inventory 9 and 10 items. The minimum and maximum values range from 0 to 10, with higher score represents higher higher frequency of experiencing particular affect.<br><br>• Psychological Well-being- Spirituality ('change' is being assessed) [Time Frame: Change from baseline Spirituality at post intervention (8 weeks), 36-week gestation and 5-week postpartum.]

	Spirituality is measured by Chinese Daily Spiritual Experience Sacle - 16 items. The minimum and maximum values range from 0 to 5, with higher score represents higher frequency of experiencing spirituality.<br>• Physical well-being-Stagnation ('change' is being assessed) [Time Frame: Change from baseline Stagnation at post intervention (8 weeks), 36-week gestation and 5-week postpartum.]<br>Stagnation is measured by Stagnation Scale - 16 items. The minimum and maximum values range from 0 to 10, with higher score represents higher stagnation level.
